# Efficacy and safety of electroacupuncture for secondary constipation: a systematic review and meta-analysis

**DOI:** 10.1007/s00384-023-04487-6

**Published:** 2023-07-15

**Authors:** Liu Jie, Liu Shiping, Xing Yue, Zhang Fuli

**Affiliations:** 1 Heilongjiang University of Traditional Chinese Medicine, Heilongjiang, 150000 China; 2 Heilongjiang Academy of Traditional Chinese Medicine, Heilongjiang, 150000 China

**Keywords:** Electroacupuncture, Constipation, Meta-analysis, Systematic review

## Abstract

**Objective:**

Secondary constipation refers to constipation that occurs after certain diseases or medications, such as acute stroke or opioids, and the efficacy of electroacupuncture for secondary constipation is controversial. So, this study aimed to explore the efficacy and safety of electroacupuncture for secondary constipation through a meta-analysis and systematic review.

**Method:**

We retrieved articles from PubMed, Embase, Cochrane Library, Web of Science, CNKI, Wanfang, and VIP databases up to 28 February 2023. The study was screened strictly according to inclusion and exclusion criteria. Revman5.4 was used for quality evaluation; grade rating was used for index evaluation, and stata15.0 was used for data consolidation analysis.

**Result:**

Thirteen randomized controlled studies, involving a total of 1437 people (722 electroacupuncture and 715 control groups), were included in this review. Meta-analysis results indicated that electroacupuncture significantly improved constipation overall response (RR = 1.31, 95%CI: 1.11, 1.55, *P* < 0.001), reduced defecation straining score (MD =  − 0.46, 95%CI: − 0.67, − 0.251, *P* < 0.001), increased weekly complete spontaneous bowel movements (MD = 0.41, 95%CI: 0.20, 0.63, *P* = 0.002), and increased in the weekly spontaneous bowel movements (MD = 0.80, 95%CI (0.49, 01.11), *P* < 0.001), and electroacupuncture had no effect on change stool consistency score compared (MD =  − 0.03, 95%CI (− 0.38, 0.33), *P* = 0.88) and did not increase adverse events (RR = 0.50, 95%CI: 0.18, 1.44, *P* = 0.20).

**Conclusion:**

According to the current studies, the overall relief rate of patients with secondary constipation after electroacupuncture treatment was improved, the defecation pressure score was reduced, the weekly natural defecation was more complete, and adverse reactions were not increased. Electroacupuncture therefore shows potential for treating constipation, but more high-quality studies are needed to confirm these findings.

**Supplementary Information:**

The online version contains supplementary material available at 10.1007/s00384-023-04487-6.

## Introduction

Constipation is a widespread gastrointestinal disorder, affecting individuals worldwide [1]. With the aging population and changes in lifestyle, it is projected that the prevalence of constipation will continue to rise, leading to an increase in disease burden and impact on work and life [2, 3]. Chronic constipation rates range from 3.2 to 45.0% in North America, Europe, Asia, Oceania, South America, and South Africa [4, 5]. Contributing factors to the high incidence of constipation include insufficient fluid and dietary fiber intake, reduced daily physical activity, reduced social support, poor health, and comorbidities such as diabetes, stroke, and Parkinson’s disease [6, 7]. Adverse drug reactions may also contribute to the onset of constipation. Recurrent constipation can reduce patients’ quality of life and lead to non-gastrointestinal complications such as hypertension and acute cardiovascular and cerebrovascular diseases from forced defecation, as well as gastrointestinal complications such as gastritis, gastroesophageal reflux, and hemorrhoids [8]. Secondary constipation is classified as secondary to certain medications such as opioids or diseases such as stroke and Parkinson’s disease [9].

Western medicine currently employs gastrointestinal motion-promoting, cathartic drugs, and enema as symptomatic treatment [10]. Lactulose, polyethylene glycol, and popping dew are commonly used for drug treatment, but these drugs only offer short-term relief and may result in drug dependence, reduced autonomous defecation ability, and altered physiological function of the colon, leading to water and electrolyte imbalances, colon melanosis, and other adverse reactions. Furthermore, cathartic agents can impact the enteric nervous system’s function and disrupt its dynamic balance, potentially leading to central nervous system damage in severe cases [11–13]. Complementary medicine, such as acupuncture therapy, is widely recognized as an effective treatment for constipation [14, 15]. Electroacupuncture enhances the effects of acupuncture through electrical stimulation, increasing the circulation of qi and blood, promoting gastrointestinal peristalsis, and aiding stool excretion [16, 17]. However, the efficacy and safety of electroacupuncture for secondary constipation remain controversial [18]. This study aims to resolve these disputes and provide a new option for the clinical treatment of secondary constipation.

## Methods

The protocol has been registered in the International Prospective Register of Systematic Reviews database (PROSPERO: CRD42023403707).

### Retrieval strategy

A systematic literature search was conducted to identify randomized controlled trials on the use of electroacupuncture for the treatment of constipation. PubMed, Embase, Cochrane Library, Web of Science, CNKI, Wanfang, and VIP databases were searched for articles published as of 28 February 2023. The search terms used were “electroacupuncture” and “constipation.” Supplementary materials S1 provide details on the specific search strategies used.

### Inclusion and exclusion criteria

This study included adults who met the diagnostic criteria for secondary constipation (secondary certain surgeries, opioids, and acute stroke) [19] and utilized electroacupuncture as an intervention measure. The primary outcome indicators were overall response, change in defecation straining score, and changes in the weekly CSBMs (complete spontaneous bowel movement), while the secondary outcome indicators were change in CCS score (constipation scoring system), change in PAC-QOL total score, and incidence of adverse events. Only randomized controlled studies were included, while duplicate publications, those not related to the topic, replies and comments, reviews and meta-analyses, case reports, and studies without a comparison of on-clamp techniques, as well as those without sufficient data, were excluded from the analysis.

### Data extraction

Two independent evaluators conducted literature screening for data extraction. The screening process involved reading the title and abstract, as well as the full text of the literature, for those that were easy to judge. For literature that required further evaluation, the full text was downloaded and reviewed. The inclusion and exclusion criteria were strictly followed during the screening process, with the observation indicators of the two groups of studies extracted and cross-checked for consistency. Data extraction included the first author, year of publication, registration number, primary disease, sample size, and follow-up.

### Risk of bias evaluate

Two researchers independently assessed the quality of the studies included in the analysis. the Cochrane to Randomized Clinical Trials Risk of Bias Tool 2.0 (RoB2) [20] was used to assess the risk of bias. RoB2 was also paired with two independent investigators. A third investigator performed consensus if two investigators differed on the risk of bias analyzed. The evaluators examined the randomization process, deviations from expected interventions, missing outcome data, choice of outcome measures, and reported outcomes. Therefore, the studies were classified as low, moderate, or high risk of bias.

### Quality of evidence assessment

GRADE (grades of recommendations assessment, development, and evaluation) system [21] was used to evaluate the evidence quality of a single outcome indicator, which was divided into four grades: high, medium, low, and very low: High, further study is unlikely to change the confidence of the efficacy assessment; medium, further study is likely to affect the confidence of the efficacy assessment and may change the assessment; low, further research is highly likely to affect the confidence of the efficacy assessment, and the assessment is likely to change; very low, the outcome of any evaluation of efficacy is very uncertain.

### Data analysis

The data extracted from the studies was inputted into statistical software, Revman5.4 and Stata15.0 (StataCorp, College Station, TX, USA), for analysis. Heterogeneity was tested using either the *I*^2^ value or Q statistic. *I*^2^ values of 0%, 25%, 50%, and 75% indicated no heterogeneity, low heterogeneity, medium heterogeneity, and high heterogeneity, respectively. When *I*^2^ was ≥ 50%, sensitivity analysis was performed to explore potential sources of heterogeneity. In cases where heterogeneity was less than 50%, the fixed effects model was utilized. Additionally, the funnel plot was utilized to assess publication bias.

## Result

### Literature screening

**Fig. 1:**
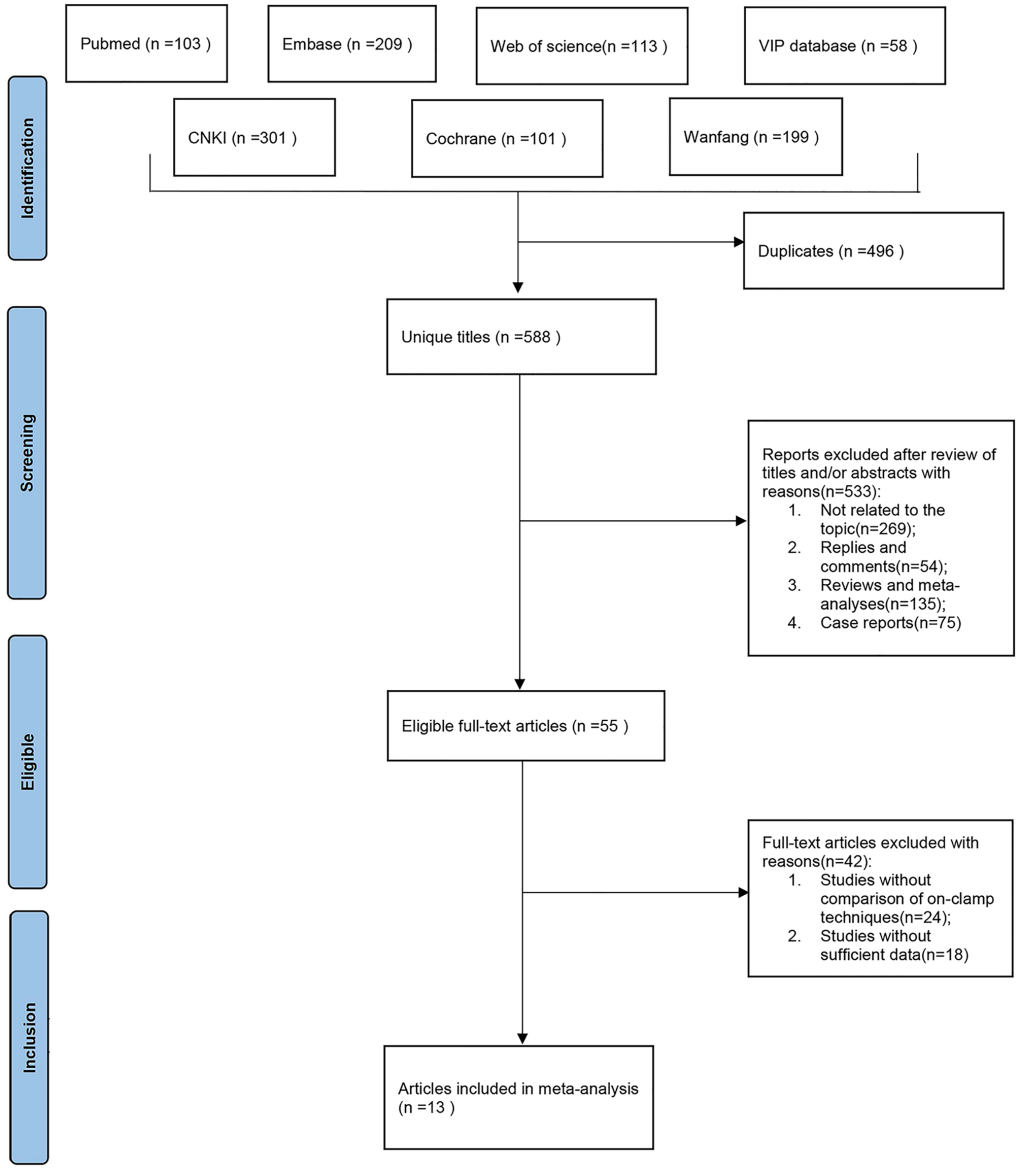
Literature search chart

A total of 604 articles were preliminarily retrieved, 464 articles were acquired after removing duplicates, 32 articles were obtained by checking the titles and abstracts of the articles, and 13 articles [22–34] were finally included in the analysis by reading the full text. See Fig. 1.

### The basic characteristics table of included literature

A total of 13 randomized controlled studies were included, involving a total of 1437 people, including 722 electroacupuncture (E) and 715 control groups (C). The specific features of the paper are shown in Table 1.

**Table 1:** Baseline characteristics of the included RCTs

Study	Study period	Registration number	Intervention	Control	Patient population	Patients (n)	Gender (male)	Age (mean/years)	Follow-up
						Electroacupuncture/control	Electroacupuncture/control	Electroacupuncture/control	
Cui 2004	-	-	Electroacupuncture	Standard medical therapy	Stroke patients	30/30	21/23	58.96/56.25	1 week
Li 2023	2018–2019	ChiCTR1800019517	Electroacupuncture + conventional pharmacological treatment	Conventional pharmacological treatment	Parkinson’s disease	83/83	45/43	67.35/66.9	24 weeks
Liu 2008	-	-	Electroacupuncture	Standard medical therapy	Stroke patients	35/35	19/21	59.6/58.9	4 weeks
Liu 2018	2013–2016	-	Electroacupuncture + conventional pharmacological treatment	Conventional pharmacological treatment	Parkinson’s disease	48/48	28/25	65.28/64.58	1 week
Sun 2010	2007–2009	-	Electroacupuncture + conventional pharmacological treatment	Conventional pharmacological treatment	Stroke patients	30/30	16/15	63.2/62.6	2 weeks
Wang 2008	2006	-	Electroacupuncture	Standard medical therapy	Stroke patients	40/40	22/23	63.2/61.9	2 weeks
Wang 2015	2012–2013	-	Electroacupuncture + conventional pharmacological treatment	Conventional pharmacological treatment	Stroke patients	170/170	82/84	63/62	8 weeks
Wang 2019	2011–2015	-	Electroacupuncture + conventional pharmacological treatment	Conventional pharmacological treatment	Stroke patients	49/49	27/31	44/43	4 weeks
Wang 2023	2019–2021	NCT03797586	Electroacupuncture	Sham electroacupuncture	Cancer patients taking opioids	50/50	29/27	63.6/65.1	8 weeks
Zhang 2009	2007–2008	-	Electroacupuncture	Standard medical therapy	Cancer patients taking morphine sulfate	33/33	17/19	59.61/63.55	2 weeks
Zhang 2010	2007–2008	-	Electroacupuncture	Standard medical therapy	Diabetic patients	50/48	30/26	59.00/57.00	1 month
Zheng 2021	2017–2020	ChiCTR-ONC-17010842	Electroacupuncture	Sham electroacupuncture	Patients with antipsychotic-related constipation	67/63	18/21	36.36/39.22	12 weeks
Zou 2020	2017–2019	-	Electroacupuncture + standard supportive care	Standard supportive care	Stroke patients	37/36	20/17	53.74/54.81	2 months

### Risk of bias assessment

**Fig. 2:**
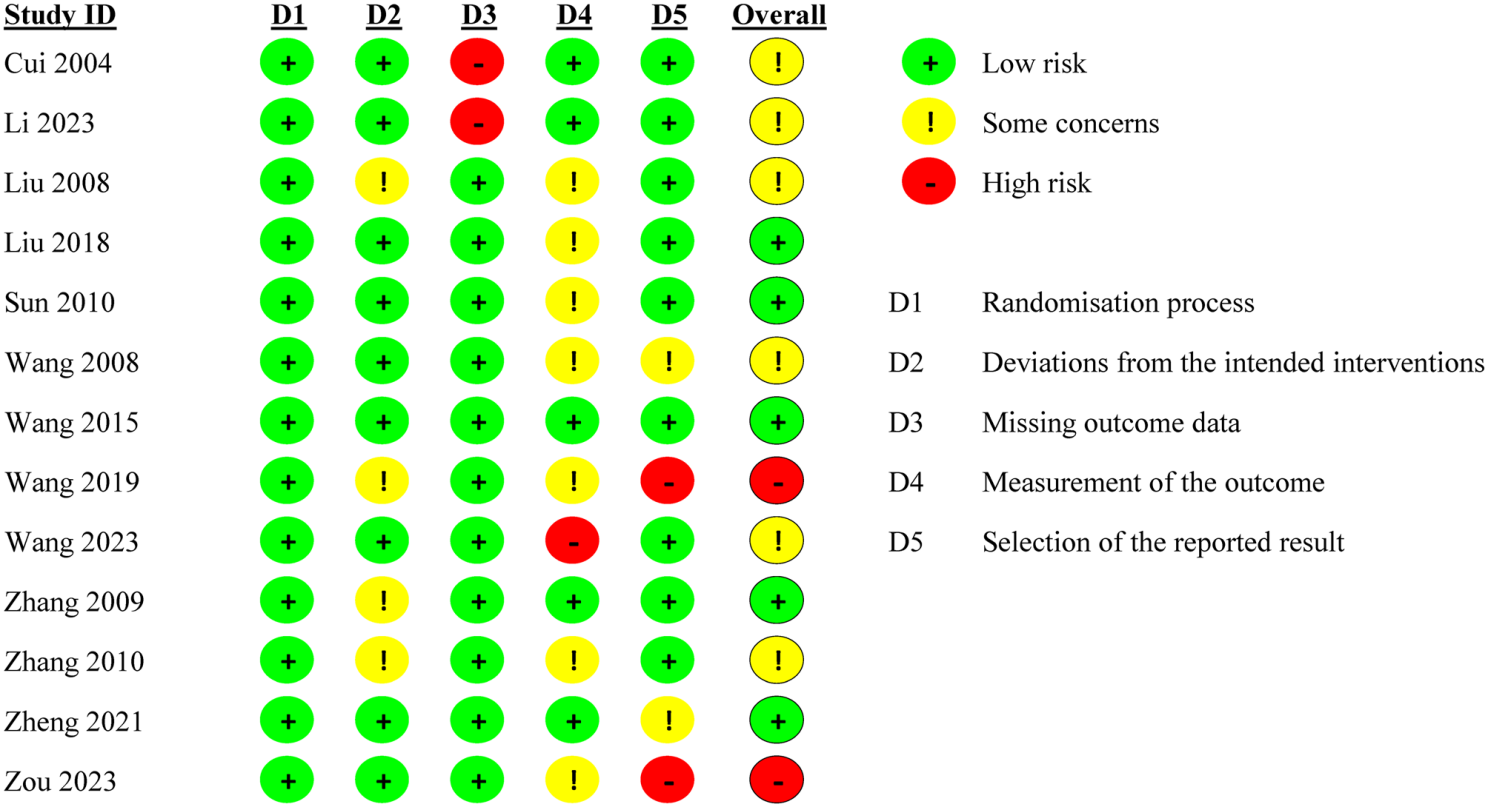
Risk bias of graph

**Fig. 3:**
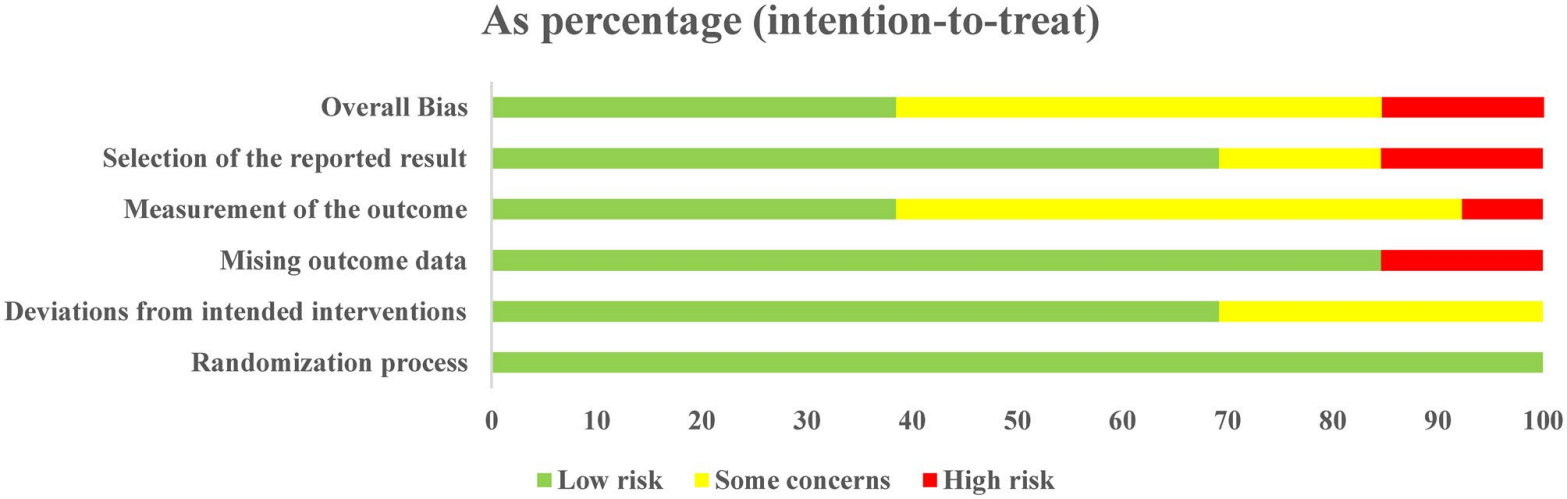
Risk bias of summary

The 13 articles included in this study all explained the method of randomization and the blind method used, and 6 articles [22, 24, 28–30] explained the blind method used for outcome evaluators. The bias risk of the articles is shown in Figs. 2 and 3.

### Meta analysis of the overall response

**Fig. 4:**
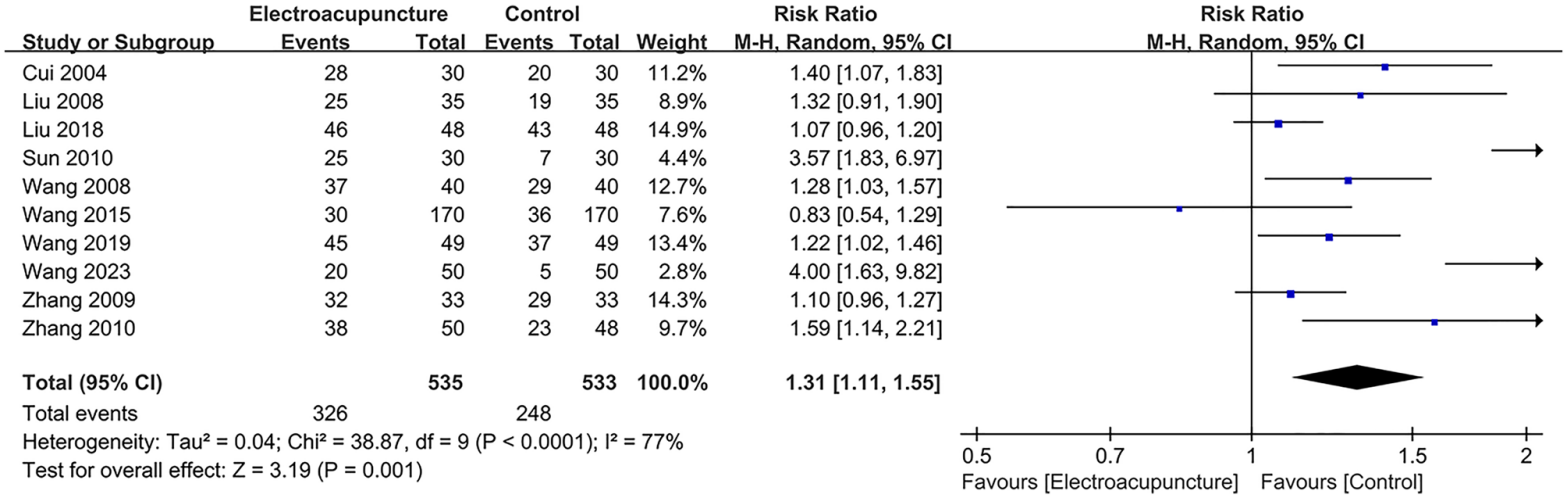
Meta analysis of overall response

**Fig. 5:**
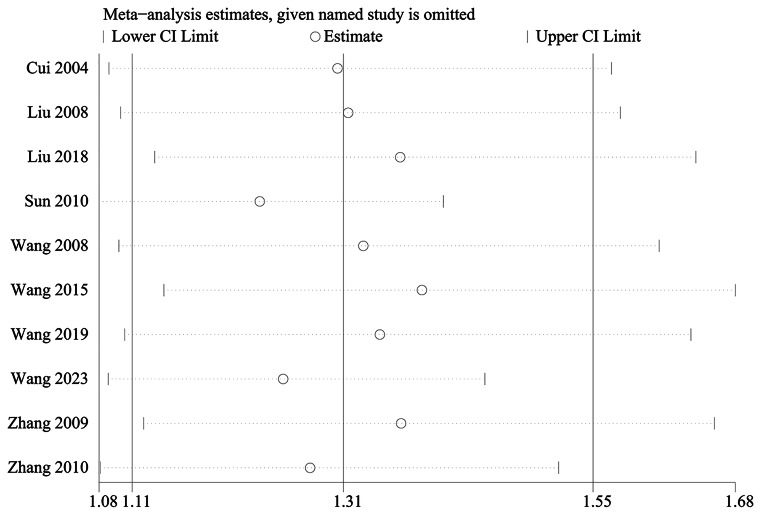
Sensitivity analysis of overall response

The overall response is defined as follows: Cure (frequency of defecation to return to previous defecation habits, or once every 1 to 3 days, soft shape, defecation time does not exceed 5 min, colon transport test within 72 h of the excretion markers more than 80%) + improvement (bowel frequency and defecation time improved than before, colon transport test within 72 h of the excretion markers improved than before) was mentioned in 10 studies, including 535 in the electroacupuncture group and 533 in the control group. A heterogeneity test (*I*^2^ = 77%, *P* < 0.001), random effects model was used for data analysis. The analysis results (RR = 1.31, 95%CI (1.11, 1.55), *P* < 0.001) suggested that electroacupuncture could improve the overall response compared with the control group, Fig. 4. As *I*^2^ > 50%, sensitivity analysis was conducted on the indicator, and the analysis result indicated that the sensitivity was small and the result was relatively stable, Fig. 5.

### Meta analysis of change in defecation straining score

**Fig. 6:**
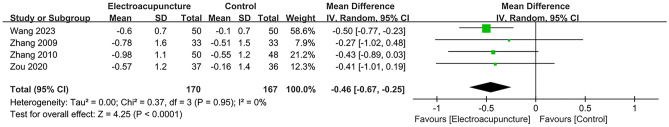
Meta analysis of change in defecation straining score

A change in defecation straining score was mentioned in 4 studies, including 170 in the electroacupuncture group and 167 in the control group. A heterogeneity test (*I*^2^ = 0%, *P* = 0.95), random effects model was used for data analysis. The analysis results (MD =  − 0.46, 95%CI (− 0.67, − 0.25), *P* < 0.001) suggested that electroacupuncture could reduce the constipation defecation straining score compared with the control group, Fig. 6.

### Meta analysis of changes in the weekly CSBMs

**Fig. 7:**
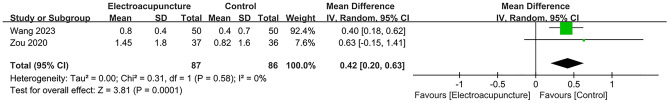
Meta analysis of changes in the weekly CSBMs

Changes in the weekly CSBMs were mentioned in 2 studies, including 87 in the electroacupuncture group and 86 in the control group. A heterogeneity test (*I*^2^ = 0%, *P* = 0.61), random effects model was used for data analysis. The analysis results (MD =  − 0.42, 95%CI (0.20, 0.63), *P* = 0.001) suggested that the electroacupuncture group had a significant increment in the weekly CSBMs compared with the control group, Fig. 7.

### Meta analysis of change in CCS score

**Fig. 8:**
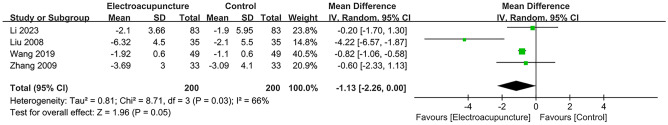
Meta analysis of change in CCS score

**Fig. 9:**
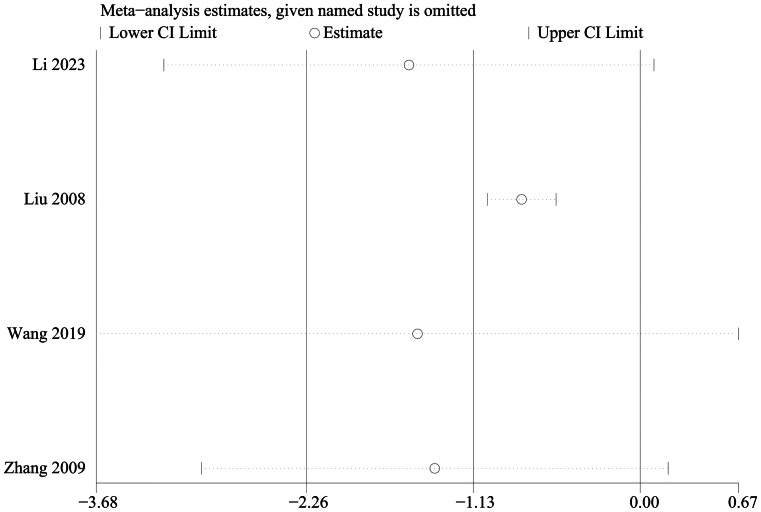
Sensitivity analysis of change in CCS score

Change in CCS score was mentioned in 4 studies, including 200 in the electroacupuncture group and 200 in the control group. A heterogeneity test (*I*^2^ = 66%, *P* = 0.03), random effects model was used for data analysis. The analysis results (MD =  − 1.13, 95%CI (− 2.26, 0), *P* = 0.05) suggested that the electroacupuncture had no effect on the CCS score compared with the control group, Fig. 8. As *I*^2^ > 50%, sensitivity analysis was conducted on the indicator, and the analysis result indicated that the sensitivity was small, and the result was relatively stable, Fig. 9.

### Meta analysis of change in PAC-QOL total score

**Fig. 10:**
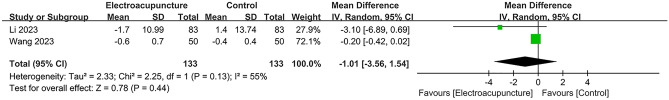
Meta analysis of change in PAC-QOL total score

Change in PAC-QOL total score was mentioned in 2 studies, including 133 in the electroacupuncture group and 133 in the control group. A heterogeneity test (*I*^2^ = 55%, *P* = 0.13), random effects model was used for data analysis. The analysis results (MD =  − 1.01, 95%CI (− 3.56, 1.54), *P* = 0.44) suggested that the electroacupuncture had no effect on PAC-QOL total score compared with the control group, Fig. 10.

### Meta analysis of adverse event

**Fig. 11:**
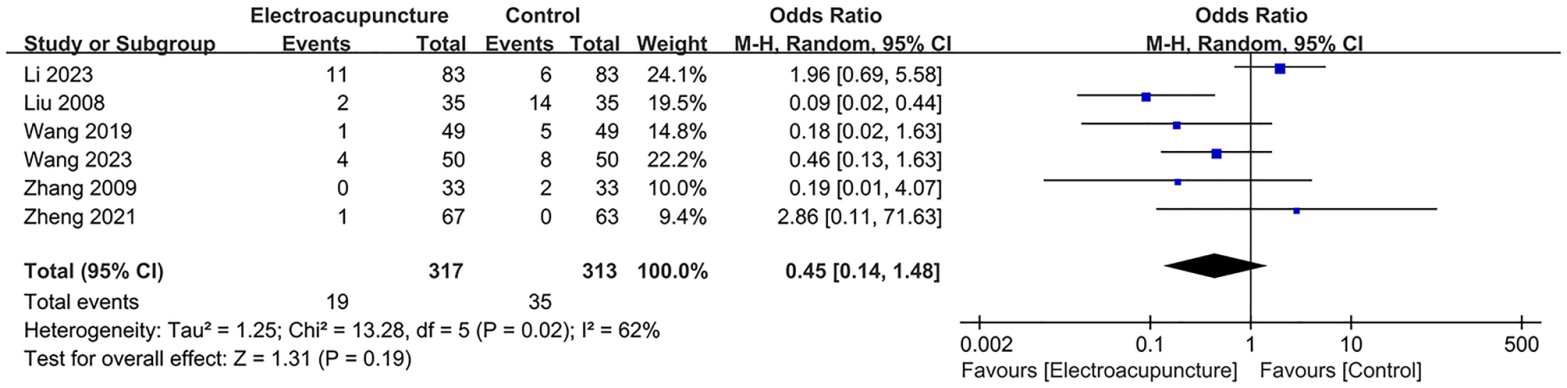
Meta analysis of adverse event

**Fig. 12:**
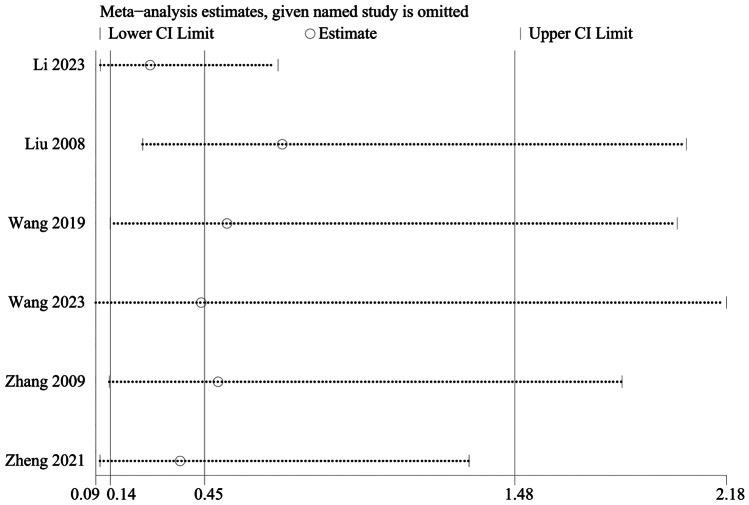
Sensitivity analysis of adverse event

An adverse event was mentioned in 8 studies, including 537 in the electroacupuncture group and 531 in the control group. A heterogeneity test (*I*^2^ = 59%, *P* < 0.001), random effects model was used for data analysis. The analysis results (RR = 0.50, 95%CI (0.18, 1.44), *P* = 0.20) suggested that compared with the control group, there was no increase in adverse event in the electroacupuncture group, Fig. 11. As *I*^2^ > 50%, sensitivity analysis was conducted on the indicator, and the analysis result indicated that the sensitivity was small, and the result was relatively stable, Fig. 12.

### Meta analysis of change in stool consistency score

**Fig. 13:**
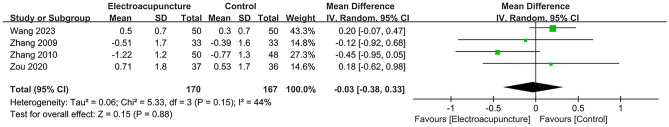
Meta analysis of change in stool consistency score

A change in stool consistency score was mentioned in a total of 4 articles, including 170 samples in the electroacupuncture group and 167 in the control group. The heterogeneity test (*I*^2^ = 44%, *P* = 0.15) was performed by random effects model. The results of meta-analysis (MD =  − 0.03, 95%CI (− 0.38, 0.33), *P* = 0.88) suggested that electroacupuncture had no effect on change in stool consistency score compared with the control group, Fig. 13.

### Meta analysis of changes in the weekly SBMs

**Fig. 14:**
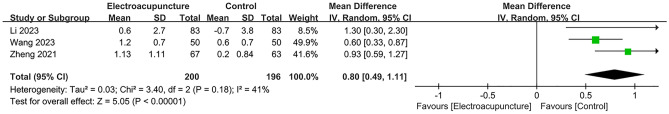
Meta analysis of changes in the weekly SBMs

Changes in the weekly SBMs was mentioned in a total of 3 articles, including 200 samples in the experimental group and 196 samples in the control group. Heterogeneity test (*I*^2^ = 41%, *P* = 0.18) was performed by random effects model. The results of the meta-analysis (MD = 0.80, 95%CI (0.49, 01.11), *P* < 0.001) suggested that the electroacupuncture could improve changes in the weekly SBMs compared with the control group, and the difference was statistically significant, Fig. 14.

### Subgroup analysis

We conducted a subgroup analysis of the overall response of the included index according to different follow-up times, different intervention measures, and different original diseases. The specific results of the subgroup analysis are shown in Table 2.

**Table 2:** Subgroup analysis of the overall response

Subgroup	Overall response			
	Study	RR [95%CI]	*P* value	*I*^2^
**Total**	10	1.31 [1.11–1.55]	0.001	77%
**Intervention**				
Electroacupuncture	6	1.32 [1.06–1.64]	0.01	83%
Electroacupuncture + conventional pharmacological treatment	4	1.36 [0.92–2.01]	0.12	77%
**Primary disease**				
Stroke	6	1.32 [1.07–1.61]	0.008	63%
Parkinson’s disease	1	1.07 [0.96–1.20]	0.24	NA
Cancer	2	2.05 [0.21–20.04]	0.54	96%
Diabetes	1	1.59 [1.14–2.21]	0.007	NA
**Follow-up**				
≥ 8 weeks	2	1.74 [0.37–8.16]	0.48	90%
< 8 weeks	8	1.31 [1.11–1.54]	0.001	78%

### Publication bias

**Fig. 15:**
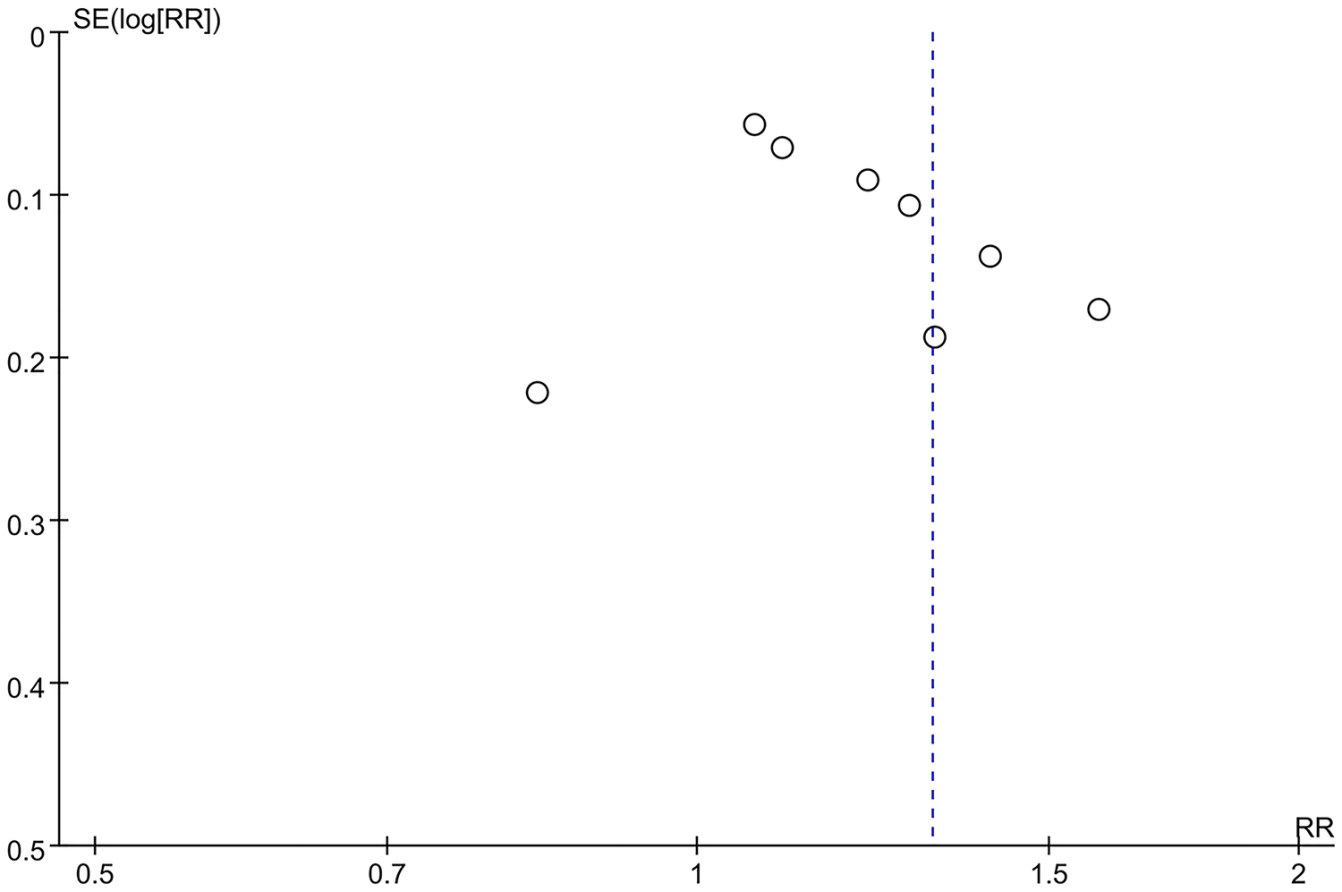
Funnel plot of overall respond

**Fig. 16:**
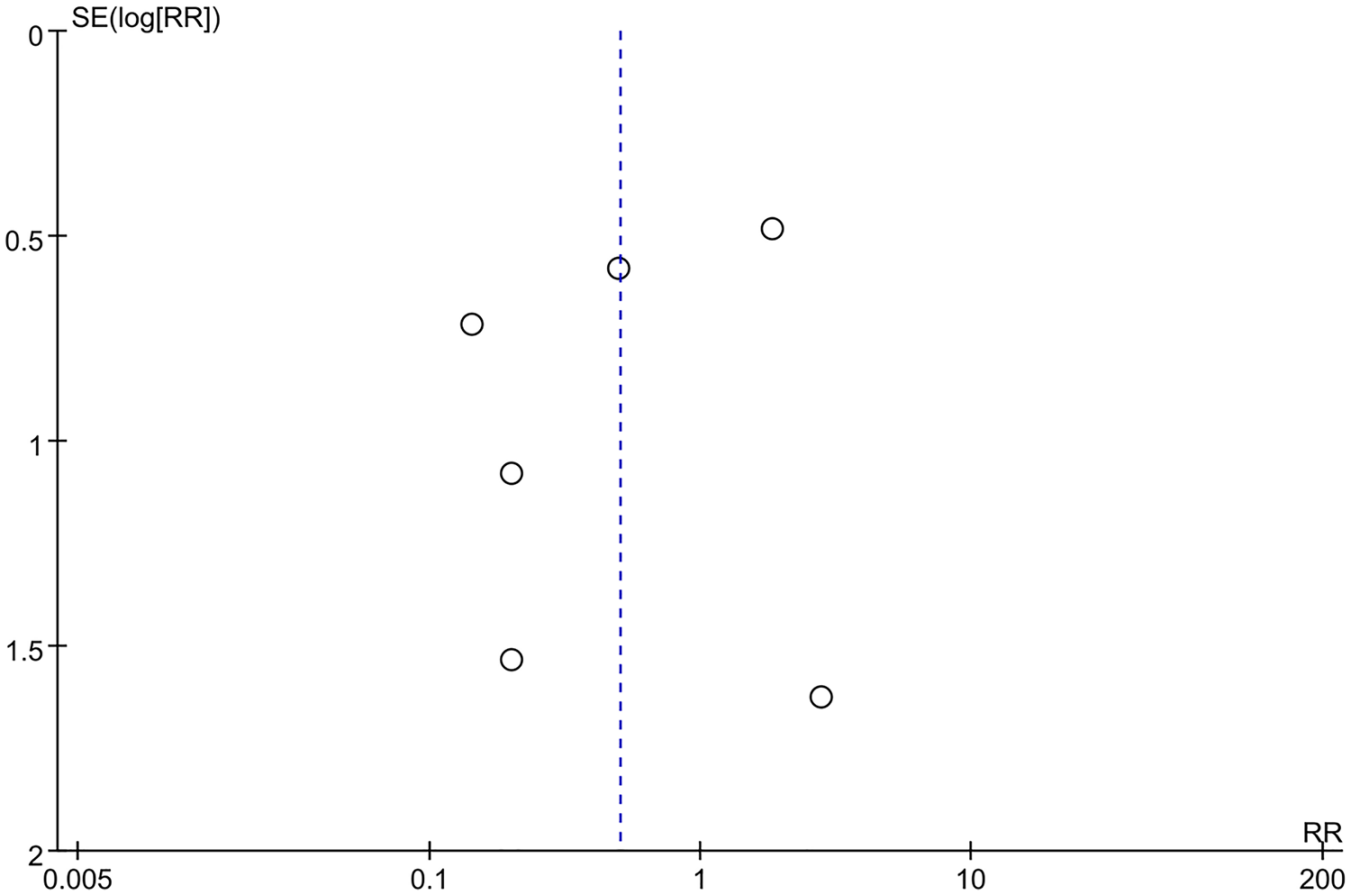
Funnel plot of adverse event

A funnel plot was used to evaluate publication deviation of the included indicators, such as adverse event and overall response. Results suggested that there is publication bias in these indicators (see Figs. 15 and 16).

### Quality of evidence assessment

According to grage, the included research indicators were rated, and the rating results showed that overall response, change in PAC-QOL total score, change in CCS score, and adverse events were all of low credibility. See Table 3 for details.

**Table 3:** Quality of evidence assessment

Outcome	Grade classification
Overall response	Low
Changes in the weekly SBMs	Moderate
Changes in the weekly CSBMs	Moderate
Change in stool consistency score (Bristol stool scale)	Moderate
Change in defecation straining score	Moderate
Change in PAC-QOL total score	Low
Change in CCS score	Low
Adverse events	Low

## Discussion

Secondary constipation is primarily caused by decreased intestinal motility [35], which can be attributed to increased sympathetic nervous tension and decreased parasympathetic excitability [36]. However, electroacupuncture has a bidirectional regulatory effect on visceral diseases. This is because the acupoints and the visceral organs they innervate form a closely connected structure-functional unit, which is controlled by the sympathetic nerve [14, 37]. Within the allosegmental innervation area of this unit, the acupoints form a functional set that can exert an opposite effect through the parasympathetic pathway. This functional set consists of two points that help maintain and regulate the homeostasis of intestinal organs. The colon contains a high concentration of neuro-endocrine cells and is also the location of the largest number of microbes in the gastrointestinal tract, making it a crucial intersection of the nervous, endocrine, and immune systems. Acupuncture of this functional set can regulate the gastrointestinal tract from multiple pathways and targets [15, 38].

According to scientific research, electroacupuncture has been found to improve the response to secondary constipation treatment. This is due to its dual effect of acupuncture and electrical stimulation, which generates electrical stimulation at abdominal acupoints to pacify intestinal slow wave and promote intestinal contraction [39]. This stimulation can also effectively overcome the phenomenon of electroacupuncture tolerance. Furthermore, electroacupuncture has been found to promote nerve fiber regeneration, which is often associated with dysplasia of nerve fiber in the colon wall, a common factor in secondary constipation [40–42]. Studies have also shown that electroacupuncture can regulate the content of brain and intestinal peptides in the body, thereby improving the brain-intestinal axis pathway and regulating intestinal function and movement. Similar to previous research, electroacupuncture combined with moxibustion at specific points can promote the recovery of intestinal flora diversity, regulate intestinal prebiotic flora, inhibit pathogenic bacteria, and restore intestinal flora stability [43]. Therefore, electroacupuncture is a beneficial treatment for constipation as it can improve the diversity and content of intestinal flora, regulate the dynamic balance of intestinal flora quality and quantity, and enhance the body’s immunity [44]. However, it should be noted that in addition to the feeling of inadequate defecation, the symptoms of secondary constipation should include the frequency of defecation, defecation effort, fecal quality, anal rectum obstruction, and the need for assisted operation with defecation, which need to be studied as a whole according to the diagnostic criteria of Rome III [45].

However, this study has several limitations. Firstly, the number of included studies is small, with most studies being from China, and few studies carefully describing the use of the blind method. The observation indicators used in well-designed foreign research reports are also different from those used in domestic studies, making it impossible to analyze them together with domestic literature, which may lead to limitations in the results. Secondly, the overall quality of the included literature is not high, and all the included studies have not been registered in the Chinese clinical trial registry, so it is impossible to judge whether the study results are selectively reported. Some of the constipation cases included in this study were due to Parkinson’s disease and others due to stroke, contributing to the high heterogeneity of our study. Moreover, due to the limited number of included studies, no subgroup analysis could be performed to explore the source of heterogeneity.

## Conclusion

According to the current study, electroacupuncture improves the overall response to constipation treatment, defecation straining score, increases weekly CSBM, and would not increase adverse effects. However, due to the limitations of this study, we hope that more high-quality, large-sample studies will be available in future studies to support our findings.

### Supplementary Information

Below is the link to the electronic supplementary material.

Supplementary file1 (DOC 24 KB)

## Data Availability

The original contributions presented in the study are included in the article, further inquiries can be directed to the corresponding author.
