# Supplementary material for: Efficacy and safety of electroacupuncture for secondary constipation: a systematic review and meta-analysis
Source: Int J Colorectal Dis. 2023 Jul 15;38(1):196. doi: 10.1007/s00384-023-04487-6 (PMC10349776; doi:10.1007/s00384-023-04487-6)
Supplement: Supplementary file 1 — Supplementary file1 (DOC 24 KB) [file 384_2023_4487_MOESM1_ESM.doc]

**Efficacy and safety of electroacupuncture for** **secondary constipation: a meta-analysis and systematic review**

**Journal name**: [INTERNATIONAL JOURNAL OF COLORECTAL DISEASE](http://www.letpub.com.cn/index.php?page=journalapp&view=detail&journalid=3695)

Liu Shiping, Liu Jie, Xing Yue, Zhang Fuli

**Liu Shiping, Liu Jie, Zhang Fuli,** Heilongjiang University of Traditional Chinese Medicine, Heilongjiang 150000, China

**Xing Yue,** Heilongjiang Academy of Traditional Chinese Medicine, Heilongjiang 150000, China

**Corresponding to：Zhang Fuli,** Professor, Heilongjiang University of Traditional Chinese Medicine, Harbin 150000, China. ljadelle@163.com

| [Website](https://s.wanfangdata.com.cn/advanced-search/paper?q=主题:(便秘) and 主题:(电针)&type=[) | [Search strategy](https://s.wanfangdata.com.cn/advanced-search/paper?q=主题:(便秘) and 主题:(电针)&type=[) |
| --- | --- |
| [PubMed](https://s.wanfangdata.com.cn/advanced-search/paper?q=主题:(便秘) and 主题:(电针)&type=[) | [(((Constipation) OR (Dyschezia)) OR (Colonic Inertia)) AND (electroacupuncture)](https://s.wanfangdata.com.cn/advanced-search/paper?q=主题:(便秘) and 主题:(电针)&type=[) |
| [Embase](https://s.wanfangdata.com.cn/advanced-search/paper?q=主题:(便秘) and 主题:(电针)&type=[) | [(](https://s.wanfangdata.com.cn/advanced-search/paper?q=主题:(便秘) and 主题:(电针)&type=[)'constipation'/exp OR constipation OR dyschezia OR (colonic AND inertia)) AND electroacupuncture |
| [Cochrane Library](https://s.wanfangdata.com.cn/advanced-search/paper?q=主题:(便秘) and 主题:(电针)&type=[) | [electroacupuncture (title abstract keyword) AND](https://s.wanfangdata.com.cn/advanced-search/paper?q=主题:(便秘) and 主题:(电针)&type=[) constipation(title abstract keyword) |
| [Web of Science](https://s.wanfangdata.com.cn/advanced-search/paper?q=主题:(便秘) and 主题:(电针)&type=[) | [Constipation](https://s.wanfangdata.com.cn/advanced-search/paper?q=主题:(便秘) and 主题:(电针)&type=[) (Topic) or Dyschezia (Topic) or Colonic Inertia (Topic) and electroacupuncture (Topic) |
| [Wanfang](https://s.wanfangdata.com.cn/advanced-search/paper?q=主题:(便秘) and 主题:(电针)&type=[) | [主题:(便秘) and 主题:(电针)](https://s.wanfangdata.com.cn/advanced-search/paper?q=主题:(便秘) and 主题:(电针)&type=[) |
| [VIP database](https://s.wanfangdata.com.cn/advanced-search/paper?q=主题:(便秘) and 主题:(电针)&type=[) | [(题名或关键词=电针 AND 题名或关键词=便秘) AND (期刊范围:核心期刊 OR OR CSCD来源期刊 OR CSSCI来源期刊)](https://qikan.cqvip.com/Qikan/search/index?LngMySearHistoryIdGuid=73d1b4ee-b40c-4080-a675-1ea40e033e51&from=Qikan_Article_History) |
| [CNKI](https://s.wanfangdata.com.cn/advanced-search/paper?q=主题:(便秘) and 主题:(电针)&type=[) | [主题](https://s.wanfangdata.com.cn/advanced-search/paper?q=主题:(便秘) and 主题:(电针)&type=[):(便秘) and(电针) |
